# Retraction Note: Acute myeloid leukemia-derived exosomes deliver miR-24-3p to hinder the T-cell immune response through DENN/MADD targeting in the NF-κB signaling pathways

**DOI:** 10.1186/s12964-025-02574-5

**Published:** 2025-12-05

**Authors:** Khalid Otmani, Redouane Rouas, Laurence Lagneaux, Mohammad Krayem, Hugues Duvillier, Mimoune Berehab, Philippe Lewalle

**Affiliations:** 1 Experimental Hematology Laboratory, Hematology Department, Hôpital Universitaire de Bruxelles, (H.U.B.) Institut Jules Bordet, Université Libre de Bruxelles, 90 Meylemeersch Street, Brussels, 1070 Belgium; 2 Laboratoire de Thérapie Cellulaire Clinique (LTCC), Institut Jules Bordet, Université Libre de Bruxelles, Brussels, Belgium; 3 Laboratory of Clinical and Experimental Oncology, Institut Jules Bordet, Université Libre de Bruxelles, Brussels, Belgium; 4 Flow Cytometry Facility, Hôpital Universitaire de Bruxelles (H.U.B.) Institut Jules Bordet, Université Libre de Bruxelles, Brussels, Belgium

**Retraction Note: Cell Communication and Signaling 21, 253 (2023)**

**https://doi.org/10.1186/s12964-023-01259-1**

The Editor-in-Chief has retracted this article after concerns were raised about the data reported. Specifically, there appears to be duplication between the following blots:

- β-actin in Figs. 3d and 4e;
- β-actin in Figs. 3e and 4b.
- Fig. 5c p-65 and Fig. 6d ERK;
- Fig. 5c P-ERK and Fig. 5e TNFR1;
- Fig. 5c ERK and Fig. 6c JAK3;
- Fig. 5d STAT3 and Fig. 5e P-65;
- β-actin in Fig. 5e and f;
- Fig. 5f ERK and Fig. 6c JAK3.

The authors did not respond to requests from the Publisher to provide raw data for the impacted figures. The Editor-in-Chief no longer has confidence in the reliability of the results and findings of this article.

Khalid Otmani did not explicitly state whether they agree or disagree with this retraction. The remaining authors did not respond to correspondence from the Publisher regarding this retraction.

